# Finding big shots: small-area mapping and spatial modelling of obesity among Swiss male conscripts

**DOI:** 10.1186/s40608-016-0092-6

**Published:** 2016-02-18

**Authors:** Radoslaw Panczak, Leonhard Held, André Moser, Philip A. Jones, Frank J. Rühli, Kaspar Staub

**Affiliations:** Institute of Evolutionary Medicine, University of Zurich, Winterthurerstrasse 190, CH-8057 Zurich, Switzerland; Institute of Social and Preventive Medicine, University of Bern, Finkenhubelweg 11, CH-3012 Bern, Switzerland; Epidemiology, Biostatistics and Prevention Institute, University of Zurich, Hirschengraben 84, CH-8001 Zurich, Switzerland; Department of Geography, Swansea University, Wallace Building, Singleton Park, Swansea, SA2 8PP UK

**Keywords:** Obesity, Switzerland, Conscripts, Disease mapping, Spatial hierarchical Bayesian analysis, Integrated nested Laplace approximation

## Abstract

**Background:**

In Switzerland, as in other developed countries, the prevalence of overweight and obesity has increased substantially since the early 1990s. Most of the analyses so far have been based on sporadic surveys or self-reported data and did not offer potential for small-area analyses. The goal of this study was to investigate spatial variation and determinants of obesity among young Swiss men using recent conscription data.

**Methods:**

A complete, anonymized dataset of conscription records for the 2010–2012 period were provided by Swiss Armed Forces. We used a series of Bayesian hierarchical logistic regression models to investigate the spatial pattern of obesity across 3,187 postcodes, varying them by type of random effects (spatially unstructured and structured), level of adjustment by individual (age and professional status) and area-based [urbanicity and index of socio-economic position (SEP)] characteristics.

**Results:**

The analysed dataset consisted of 100,919 conscripts, out of which 5,892 (5.8 %) were obese. Crude obesity prevalence increased with age among conscripts of lower individual and area-based SEP and varied greatly over postcodes. Best model’s estimates of adjusted odds ratios of obesity on postcode level ranged from 0.61 to 1.93 and showed a strong spatial pattern of obesity risk across the country. Odds ratios above 1 concentrated in central and north Switzerland. Smaller pockets of elevated obesity risk also emerged around cities of Geneva, Fribourg and Lausanne. Lower estimates were observed in North-East and East as well as south of the Alps. Importantly, small regional outliers were observed and patterning did not follow administrative boundaries. Similarly as with crude obesity prevalence, the best fitting model confirmed increasing risk of obesity with age and among conscripts of lower professional status. The risk decreased with higher area-based SEP and, to a lesser degree – in rural areas.

**Conclusion:**

In Switzerland, there is a substantial spatial variation in obesity risk among young Swiss men. Small-area estimates of obesity risk derived from conscripts records contribute to its understanding and could be used to design further studies and interventions.

**Electronic supplementary material:**

The online version of this article (doi:10.1186/s40608-016-0092-6) contains supplementary material, which is available to authorized users.

## Background

The prevalence of overweight and obesity (OWOB), with steady increase over three decades, has reached global pandemic proportions in the developed world [1–3], with over 1.4 billion adults classed as overweight in 2008. Within this figure, over 200 million men and nearly 300 million women were obese [4].

The negative associations of overweight and obesity with chronic disease such as diabetes type II, hypertension, cardiovascular disease and some cancers is well documented [5–8]. Currently there is some scientific debate regarding a possible protective effect of low-level overweight to lower mortality risk, yet the negative effect of obesity on morbidity and mortality is certain [9, 10]. Quality of life for overweight or obese adolescents and adults has been linked to poorer health outcomes, low family income and a limiting effect on marriage possibilities later in life [11, 12].

From the early 1990s an increase in the prevalence of OWOB in many developed countries including Switzerland [13–15]has imposed a burden on the population and health care system [16]. In 2006, OWOB and its co-morbidities, and health consequences constituted 11 % of total Swiss healthcare expenditure [17] including: 27,000 cases of diabetes type II, 63,000 cases of hypertension and 37,000 cases of lipid metabolism disorders. These health risks and costs could have been circumvented had OWOB remained at 1992 levels [5].

Research into OWOB in Switzerland comprises an important component for public health research and planning. However longitudinal, nationally measured data on the prevalence of OWOB in Switzerland is lacking [13]. A number of studies have provided socioeconomic and regional differences on the prevalence of OWOB but many of these were based on sporadic surveys with subsequently limiting data on regional, demographic and socio-economic representation [14, 18–24]. These studies might also be biased by sample selection/size and low participant numbers [14]. Self-reporting of stature and weight in some surveys such as the Swiss Health Survey (SHS) [25] and the Swiss Household Panel [26, 27] may also introduce bias.

These results indicate early signs of a slowdown in the increase of OWOB among both adults and children in recent years, implying some success in increased awareness of the condition and public health programmes targeting physical activity and healthy eating [22, 28–32]. Estimates of obesity prevalence varied widely, between 3 and 15 % (BMI= > 30 kg/m2), as a result of which sex, age, ethnic or socioeconomic group and which anthropometric measurement method was analysed. Variability between studies of obesity prevalence is substantial between waves of the SHS [25] and the various regional and epidemiological studies [13, 20, 33–36]. Estimates of small-area regional variability of OBOW and their determinants presently exist only for the city of Geneva and such estimates are lacking elsewhere in Switzerland [37].

The complex pathogenesis of obesity has yet to be completely understood [38]. In addition to genetic and epigenetic factors [39], environmental factors, socio-economic position, and behavioural choices such as daily food intake and energy expenditure have an impact on the risk of obesity [7]. Even public health policies like smoking bans might have unintended effects and provoke weight gain [40]. Moreover, the literature has featured a steady increase in the number of reports suggesting associations with small-area community or neighbourhood characteristics (e.g., access to playgrounds and to grocery stores with affordable healthful food), social-network effects, and home-environment factors (adequate housing, culture, parental model, etc.) on obesity [7, 41–44]. Understanding such area-based epidemiological features of obesity is an important step toward understanding its aetiology and prioritizing future public-health interventions.

The main goal of this study is to investigate small-area variation and determinants of obesity among young Swiss men using georeferenced conscription data from 2010–2012 in order to better understand spatial and socio-economic pattering of obesity in Switzerland and to assist planning interventions on a small scale.

## Methods

### Conscript data in public health research

Nations in Europe which continue to conscript their male citizens into military infrastructure include Finland, Norway, Denmark, Austria and Switzerland. Conscription is mandatory for all young males and entails a medical examination with recording of standardised anthropometric measurements. The process is repeated annually and provides valuable anthropometric data on young men of a directive age [45]. Though these conscription systems are not outlined for collection of such data per se, they have successfully served as a basis for epidemiological studies in Switzerland [29, 46, 47], Sweden [48, 49], Austria [50–52], and Germany [53]. Though limited to young males, the prevalence of conscripts’ OBOW remains a valuable research source because obesity in young adulthood increases the likelihood of being obese later in life, and the morbidity and mortality risks of men increase with advancing age [6, 54].

### Swiss conscription process

First instituted in 1875, the mandatory recruitment for the Swiss Armed Forces was renewed and expanded in 2004. Specifically, all young Swiss men are summoned for conscription during the year in which they get 19 but may request conscription before or after this year. Mandatory assessments collect data referencing anthropometric status (measured stature and body weight, rounded to integers), socioeconomic status (occupation) and place of domicile, and are required even for those who may subsequently be granted a deferral or an exemption from service. Six conscription centres (Lausanne, Sumiswald, Windisch, Rüti, Mels and Monte Ceneri) conduct professional, medically supervised assessments using uniform quality standards for technical facilities and work processes (*Bundesgesetz über die Armee und die Militärverwaltung, Militärgesetz MG*, 510.10, Art. 2; MG Art. 9, *Verordnung über die Rekrutierung VREK*, 511.11, Art. 3 and Art. 9). Measurements are continuously entered in the medical-information database of the Swiss Armed Forces (MEDISA) and are accessible to Army personnel. The resulting data reflect over 90 % of the yearly male birth cohorts, allowing analyses of variation across temporal, regional and socioeconomic strata [29, 55].

### Data sources

Anonymised individual conscription entries covering the time period of 1 January 2010 - 31 December 2012 were provided to the study authors under contractual agreement by the Swiss Armed Forces (*Logistikbasis der Armee*, LBA San). Retained information included date of birth, date of conscription, height and weight as measured by the army personnel, current occupation (free-text input), postcode of place of domicile and conscription status (first and regular appearance or re-evaluation). The Federal Directorate of Cadastral Surveying provided a spatial dataset of postcode boundaries (Release 7 - 1. May 2013), a version of the data with postal geography as of 31 March 2013.

### Data availability and ethics statement

In accordance with Swiss law (*Bundesgesetz über die militärischen Informationssysteme MIG*, BG 510.91, Art. 2, 9, 24–29) the Swiss Armed Forces have authority to provide anonymized data for academic research; where analyses are based on such anonymized and nonclinical government data, ethical approval is not required (Swiss data privacy act, SR 235.1; 19.6.1992 and Federal Act on Research involving Human Beings HRA, 810.30; 1.1.2014). Thus upon approval of the project protocol by *Logistikbasis der Armee - LBA San* [56], a signed bilateral data contract was enacted between the study authors and the Swiss Armed Forces. Names, identification numbers and residential addresses were removed prior to data delivery. As conscription in Switzerland is mandatory and the body measurements analysed in this publication fit the profile of nonclinical government data, informed consent is not required.

### Study population

The study included male conscripts between 18 and 22 years of age presenting for the first regular assessment in one of the recruitment centres. Individuals with a missing or incorrect postcode or insufficient information regarding occupational status were excluded as well as conscripts with implausible stature and weight values (130 cm > height > 220 cm; 30 > weight > 200 kg). Apart from one young man with a recorded weight of 500 kg, none of the individuals were above 200 kg. No unfeasible BMI values were found.

### Spatial resolution

We used postcode of place of residence at the time of conscription as the spatial resolution for the study. Because Swiss postcode boundaries change over time (due to creation or deletion, merges, splits, etc.) we standardized postcodes of places of residence from various years to the state on the 31^st^ of March 2013 in order to match them to the available spatial dataset of postcode boundaries.

### Representativeness

A precise assessment of the representativeness of the conscript population in comparison to the total population of young Swiss males is at present non-existent. However, an earlier study using similar sources [29] estimated data to be of high coverage (>90 %), despite using narrower age range of study participants (18.5–20.5 years of age).

### Variables

Obesity was defined using World Health Organization (WHO) classification as Body Mass Index (BMI = weight [kg]/height [m]^2^) ≥ 30 kg/m^2^ [57, 58]. Age at conscription was calculated based on the dates of birth and conscription, and then categorised into four one-year intervals from [18-19) to [21-22) years of age. For occupational status, a separate category for those in education was created (‘Pupils’) and other occupations were categorized as follows. Using the International Standard Classification of Occupations (ISCO-08) (as specified by the International Labour Organization [59]), the free-text entry for current occupation was converted, then aggregated into three major, hierarchical groups [60]: ‘Low’ (ISCO major groups 7–9), ‘Medium’ (ISCO major groups 3–6) and ‘High’ (ISCO major groups 1-2) [29].

Postcodes were classified into three categories (‘Urban’, ‘Peri-urban’ and ‘Rural’) according to the degree of urbanization of the community in which they were located using classification of the Swiss Federal Statistical Office. Finally, median value of the Swiss neighbourhood index of socioeconomic position (Swiss-SEP; [61]) was calculated for all postcodes and matched to the individual entries. Median postcode Swiss-SEP was then categorized into quintiles, in order of increasing socioeconomic status [29].

### Statistical analyses

We calculated percentages of obese conscripts across 1-year age bands, categorical variables (professional status and urbanicity) and quintiles of Swiss-SEP. Obesity was the binary outcome in all regression models. We used Bayesian hierarchical logistic regression models to investigate the spatial pattern of obesity across postcodes [62–64]. Since our main hypothesis was that obesity geographically varies across postcodes, we developed a series of models that differed in use of the unstructured and spatially structured random components and the level of adjustment in order to investigate the influence of the spatial dependence between postcodes: 1) a spatially unstructured component (i.e. area-independent effect) and no covariates (Model 1); 2) Model 1 with adjustment covariates (Model 2); 3) a spatially unstructured component and a spatially structured component (i.e. spatial effect depending on neighbouring postcodes; modelled using intrinsic conditionally autoregressive (iCAR) model) [65] without covariates (Model 3); 4) Model 3 with adjustment covariates (Model 4). CAR models in Bayesian framework allowed us to incorporate measures of connectivity between regions allowed to smooth the relative risk estimate in given area towards the mean risk in the neighbouring regions. Such approach of ‘borrowing information’ from adjacent areas allows obtaining more reliable risk estimates in comparison to using the crude rates, particularly for regions with small count of events [66]. Adjusted models used two individual (age and professional status) and two area-based characteristics (urbanicity and Swiss-SEP). In additional models we compared use of 1 year age bands and quintiles of Swiss-SEP with continuous variables and tested for potential influence of the recruitment centre. Similarly to other studies [63, 67–69], unstructured and structured random effects were modelled as independent zero-mean Gaussian random variables with vague gamma (1, 0.01) and gamma (1, 0.001) priors on the corresponding precision parameters. For the fixed effects we used zero-mean Gaussian priors with variance 1000. We used the deviance information criterion (DIC) for comparing model fits [70].

We used ‘queen’ contiguity-based spatial weights where all postcodes sharing any length of the border (including corners) to conceptualize neighbourhood relationships [71]. All analyses were done in R 3.1.1 [72] using the R- INLA library [[69], www.r-inla.org]. INLA implements the integrated nested Laplace approximation approach for latent Gaussian models [69].

Results are presented as combined proportional symbol [73] and choropleth maps. Squares placed at the median population centre of each postcode represent its estimated odds ratios (ORs) with hue, their size scaled according to the square root of the total number of conscripts from 2010–12 period. Population, and proportionally with it – conscripts, is very unevenly distributed in Switzerland, with large areas of the Alps having few or no residents. Proportional symbol mapping approach assured that postcodes with large area and sparse conscript numbers are less prominent on the map. Bayesian posterior probabilities of obesity for each postcode are shown in the same maps as an underlying layer, in shades of green. Darker hues were chosen for postcodes with higher probability. Both colour schemes were derived from ColorBrewer.org tool [[74], colorbrewer.org]. Diverging colour scheme for ORs was modified by skipping middle, light hues to improve map readability.

## Results

### Study population and crude obesity prevalence across postcodes

The initial sample consisted of 123,357 conscripts. We excluded 802 (0.7 %) women, 4,549 (3.7 %) conscripts appearing for reassessment, five (<0.1 %) with implausible values of height and weight, one (<0.1 %) with missing information of postcode of place of residence, 16 (<0.1 %) below 18 and 6,690 (5.4 %) above 22 years old and 10,375 (8.4 %) with missing or imprecise occupation. The final sample consisted of 100,919 conscripts, out of which 5,892 (5.8 %) were obese.

On 31.03.2012 there were 3,187 postcodes in Switzerland. The mean number of conscripts in postcode was 31.7 (standard deviation: 44.9) and ranged from zero (in 102 postcodes) to 505 (one postcode). Due to small population sizes there was a great variation in crude percentage of obese conscripts across postcodes ( rang from 0.0 to 100.0 %).

### Obesity prevalence across socioeconomic characteristics

**Fig. 1:**
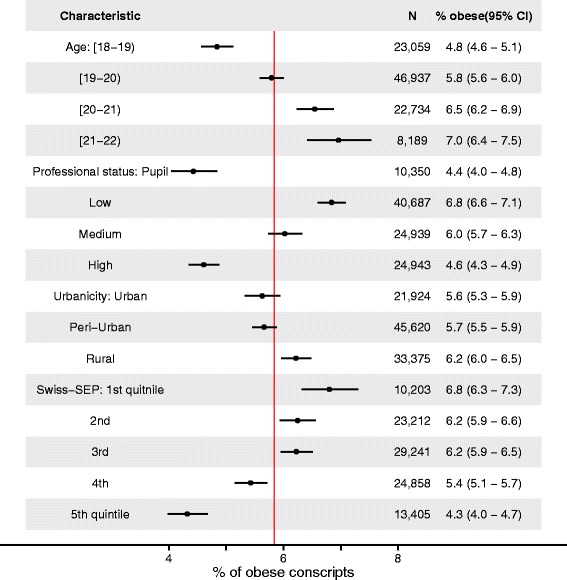
Crude prevalence of obesity across age and socioeconomic characteristics of conscripts, Switzerland 2010–12. Vertical red solid line indicates 5.8 % overall proportion of obesity

Obesity prevalence varied across sociodemographic characteristics of conscripts and by the types of areas they came from. Figure 1 shows crude percentages and their 95 % confidence intervals (CI) of obesity prevalence. There was a clear gradient of increasing percentage of obese individuals across 1 year age groups: There were 7.0 % (6.4–7.5 % 95 % CI) obese conscripts among 21 year old conscripts as opposed to 4.8 % (4.6–5.1 % 95 % CI) among 18 year olds. The opposite gradient of similar strength was observed across strata of professional status with 4.6 % (4.3–4.9 % 95 % CI) obese conscripts in the ‘High’ group as opposed to the ‘Low’ 6.8 % (6.6–7.1 % 95 % CI). A similar inverse gradient existed between 1^st^ (highest SEP) and 5^th^ (lowest SEP) quintiles of neighbourhood SEP index. Differences between conscripts coming from communities of different urbanicity levels were less pronounced.

### Obesity prevalence across postcodes

**Fig. 2:**
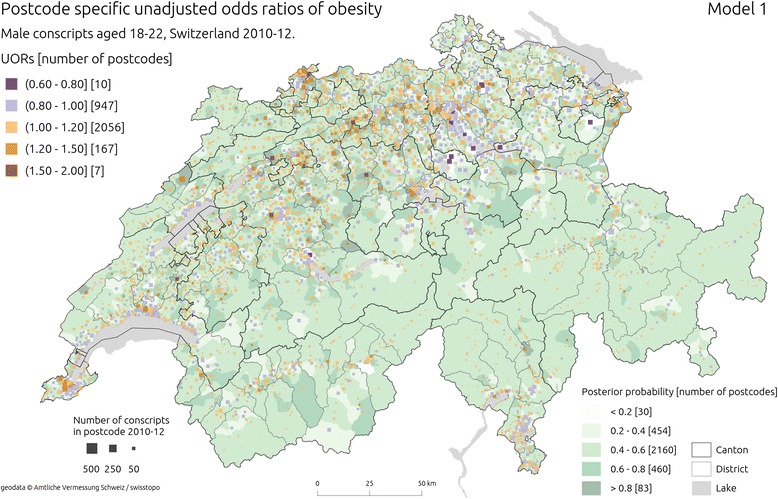
Estimated unadjusted odds ratios (UORs) of obesity from Model 1 ( using spatially unstructured effects). The squares are placed in the median population centres of the postcodes from which conscripts came from; the hue of the square is shaded according to the estimated OR in a given postcode, and its size is proportional to the square root of the number of conscripts from 2010–12 period. Background choropleth map (green hues) represents posterior probability of an increased risk of obesity. Sources of geodata: Amtliche Vermessung Schweiz/swisstopo

**Fig. 3:**
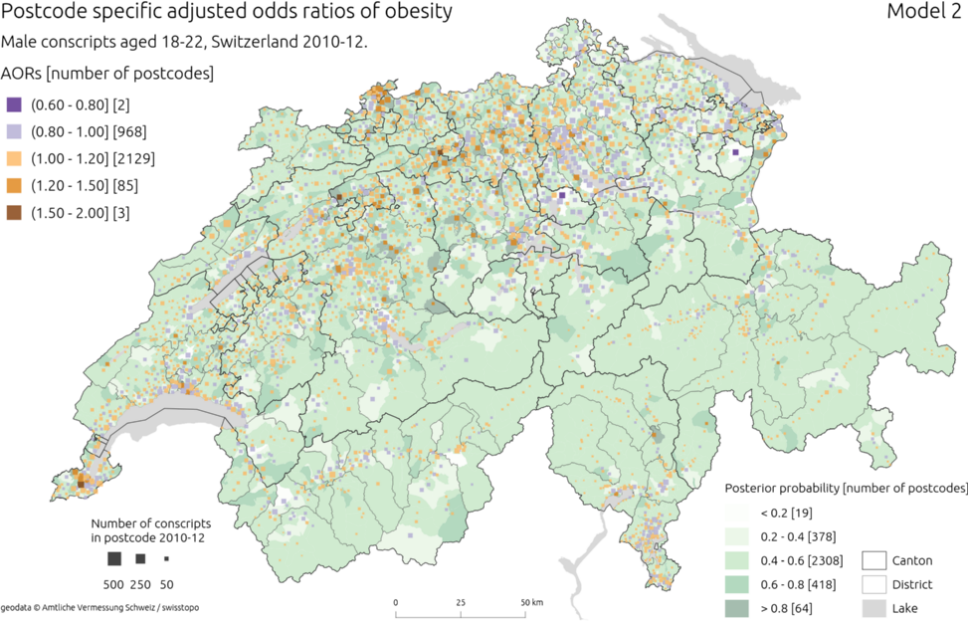
Estimated adjusted odds ratios (AORs) of obesity from Model 2 ( using spatially unstructured effects). The squares are placed in the median population centres of the postcodes from which conscripts came from; the hue of the square is shaded according to the estimated OR in a given postcode, and its size is proportional to the square root of the number of conscripts from 2010–12 period. Background choropleth map (green hues) represents posterior probability of an increased risk of obesity. Sources of geodata: Amtliche Vermessung Schweiz/swisstopo

Figure 2 shows postcode level estimates of ORs of obesity among conscripts from unadjusted (Model 1) and Fig. 3 shows the adjusted model with spatially unstructured component (Model 2). Adjustment for individual and area level covariates did not cause major changes in the spatial patterning of estimates. In both models there was a heterogeneity of ORs between postcodes. Some large scale spatial patterns emerged from the maps indicating SW-NE belt of elevated OR s in Central and North Switzerland (stretching mainly throughout postcodes located in the cantons of Basel Stadt and Basel Land, Solothurn, Aargau and northern parts of Bern and Zurich) [75]. Postcodes with ORs below 1.0 were concentrated in NE Switzerland (the cantons of Zurich and Thurgau) and scattered through the rest of the country. Posterior probability estimates were relatively low throughout whole country. DIC indicated Model 2 as having better fit compared to Model 1 (Table 1).

**Table 1:** Odds ratios across the covariates (fixed effects) together with their 95 % credible interval and model parameters of four hierarchical logistic regression models. See text for the detailed description of differences between models

Variable	Category	Model 1	Model 2	Model 3	Model 4
Age	(per 1 year)	-	1.17 (1.13–1.21)	-	1.18 (1.14–1.22)
Professional status	Pupil	-	0.75 (0.67–0.84)	-	0.70 (0.63–0.79)
	Low	-	1.10 (1.03–1.17)	-	1.10 (1.03–1.17)
	Medium	-	1.00 (ref.)	-	1.00 (ref.)
	High	-	0.73 (0.68–0.79)	-	0.74 (0.68–0.80)
Swiss-SEP	(per 10 units)	-	0.83 (0.80–0.87)	-	0.79 (0.75–0.84)
Urbanicity	Urban	-	0.97 (0.89–1.06)	-	1.01 (0.93–1.09)
	Peri-urban	-	1.00 (ref.)	-	1.00 (ref.)
	Rural	-	0.91 (0.84–0.98)	-	0.90 (0.83–0.98)
Variance of random effects	Total	0.073	0.057	0.086	0.074
	Spatial component	-	-	0.065	0.065
DIC		44,812.27	44,511.27	44,696.30	44,358.98
pD		308.59	260.16	214.66	150.35

Abbreviations*: ref. * reference category, *DIC* deviance information criterion, *pD* effective number of parameters

**Fig. 4:**
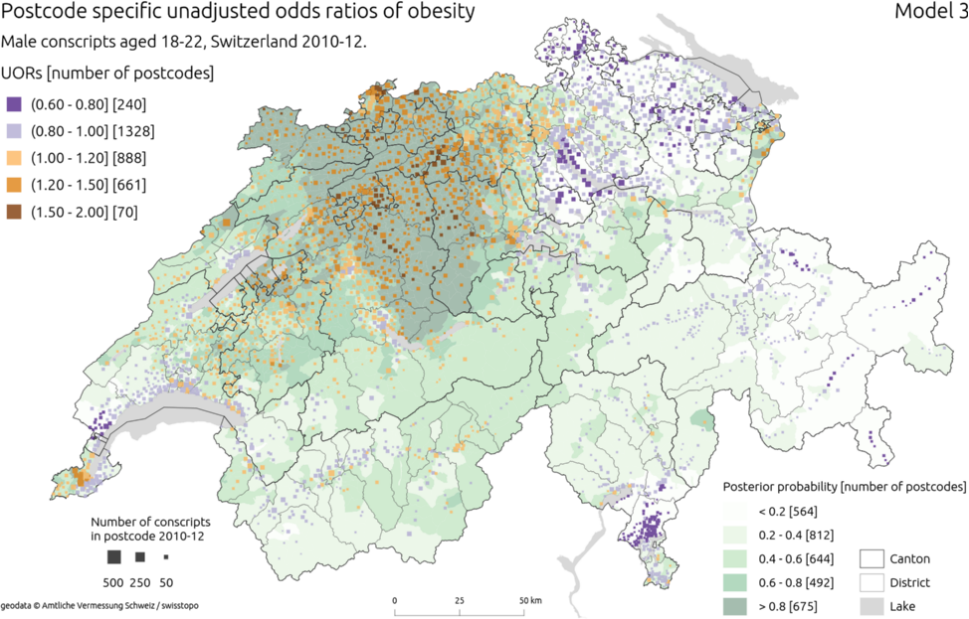
Estimated unadjusted odds ratios (UORs) of obesity from Model 3 ( using spatially unstructured and structured effects). The squares are placed in the median population centres of the postcodes from which conscripts came from; the hue of the square is shaded according to the estimated OR in a given postcode, and its size is proportional to the square root of the number of conscripts from 2010–12 period. Background choropleth map (green hues) represents posterior probability of an increased risk of obesity. Sources of geodata: Amtliche Vermessung Schweiz / swisstopo

**Fig. 5:**
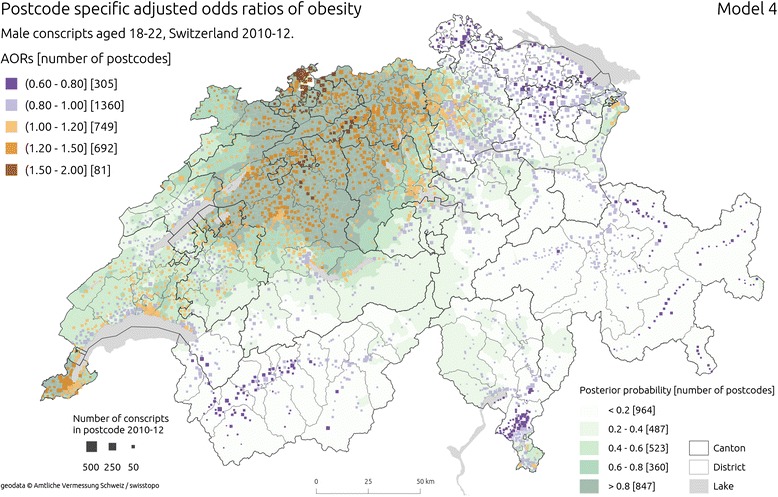
Estimated adjusted odds ratios (AORs) of obesity from Model 4 (AOR), (using spatially unstructured and structured effects) . The squares are placed in the median population centres of the postcodes from which conscripts came from; the hue of the square is shaded according to the estimated OR in a given postcode, and its size is proportional to the square root of the number of conscripts from 2010–12 period. Background choropleth map (green hues) represents posterior probability of an increased risk of obesity. Sources of geodata: Amtliche Vermessung Schweiz/swisstopo

Figure 4 shows postcode level estimates of ORs of obesity from unadjusted (Model 3) and Fig. 5, the adjusted model with spatially unstructured and structured components (Model 4). Similarly as in two previous models, adjustment for individual and area level covariates did not cause major changes in the spatial patterning of estimates. The heterogeneity of ORs increased with postcode-level estimates ranging from 0.61 to 1.93, further accentuating strong spatial structure of the results. There was a clear concentration of ORs above 1 in central and north Switzerland. Additional, smaller pockets of postcodes with higher ORs emerged around cities of Geneva, Fribourg and Lausanne. Lower estimates of ORs were observed in the North-East (cantons Thurgau, Schaffhausen, St. Gallen, Appenzell Ausserrhoden, Appenzell Innerrhoden) and East (canton Grisons) Switzerland as well as south of the Alps (in the cantons of Valais and Ticino). In contrast to the results from models without structured components, posterior probability estimates were higher in North, Central and Western part of the country.

### Comparison of the models

DIC indicated Model 4 as having best fit to the data (Table 1). Adding the spatial component helped to explain the variation in the data (more total variance), whereas adding covariates reduced the total variance (0.016 difference between Model 1 and 2, and 0.012 - between 3 and 4), but not really the spatial variance (0.001 difference between Model 3 and 4) (Table 1). Lastly, there was a noted change in the shape of distribution of postcode specific estimates of ORs between models, particularly when the spatial effects were included (Additional file 1: Figure S1). Whereas the median of the estimates was in the 0.99–1.02 range for all four models, the IQR range increased with complexity of the models and ranged 0.99–1.08, 0.99–1.06, 0.89–1.18, 0.85–1.19 for models 1 to 4 respectively.

### Association with individual and area-based characteristics

Table 1 shows the association of the covariates (fixed effects) from adjusted models 2 and 4. There was little change between the estimates after adding the spatially structured effects. The results of the best fitting model 4 confirmed most of the effects seen in crude prevalence estimates (Fig. 1). There was a significant positive effect of age [adjusted OR 1.18 per 1 year of age; 1.14–1.22 95 % credible interval (CI)] and negative effect of individual’s professional status (adjusted OR 0.70, 0.63–0.79 95 % CI comparing ‘Low’ to ‘Medium’). The association with Swiss-SEP index (reported as per 10 units increase; the original Swiss-SEP ranges from 0 to 100) was almost equal in strength (adjusted OR 0.79, 0.75–0.84 95 % CI).

Lastly, there was little effect of urbanicity of place of residence with conscripts from rural communities having slightly lower odds of being obese. In the additional model we included random effect of the conscription centre to check for any potential differences between them, but failed to find any association (results not shown).

## Discussion

The aim of this study was to describe small-area variation and socioeconomic determinants of obesity among young men in Switzerland using recent waves of conscription data. Using records from 2010–12 period, we found a strong spatial structure of risk of obesity across postcodes. A clear concentration of high and low values of ORs emerged, but also small, regional outliers were observed. Importantly, spatial patterning did not always follow administrative boundaries of large administrative units [cantons and districts (*Bezirke*)] and would be missed or distorted if they were used for the analysis. Lastly, results indicated age and socioeconomic gradients in obesity risk, with smaller influence of urbanicity.

The main strength of this study is its reliance on representative, large and objectively measured georeferenced data. The sample size allowed high-resolution analysis on the level of individual postcodes. Such findings could contribute to the understanding of the small-area obesity risk that not necessarily follows administrative boundaries (cantons) and thus help to design further studies and target interventions. The study however has several limitations and any generalisation of the results should take them into account. First of all, this study focusses on young, Swiss men only and the associations cannot be directly applied to women, other age groups and non-Swiss individuals. BMI driven obesity indicator was the only body shape measure available for this study. Because BMI cannot accurately differentiate between weight associated with fat and lean muscle mass, it not an ideal measure of body composition [76–78]. This can occasionally, lead to misclassification of physically very fit individuals, especially in the overweight BMI category 25.0–29.9 kg/m^2^. Nevertheless, BMI is strongly correlated with the body fat percentage particularly regarding the categories of obesity (BMI ≥ 30 kg/m^2^) and because of its ease of use and availability it is the most commonly utilised measure for large-scale investigations and convenient assessment in clinical practice [7, 79, 80]. By relying on standard definition of obesity, we avoided problem of ad hoc and post hoc category selection and assured comparability with other studies [81]. Additionally, profession is a limited indicator for socioeconomic background of young individuals, because a significant number of conscripts were still in education [47, 82]. We attempted to mitigate that limitation by using area-based SEP index, which at the moment is the only such indicator available in Switzerland [61]. Further, conscription data do not provide information about the migration background of the young men. Lastly, residential postcode of the conscripts was the only geographical variable available in the dataset. Swiss postcodes are still arbitrary geographical units, which were not designed for public health investigations. However, they offer a high level of resolution for national scale research whilst still maintaining privacy of individuals. Additionally, they remain in use in official health statistics as a basis for formulation of *MedStat* regions [83] and can therefore in the future be linked to other Swiss health related data such as the Swiss database of hospital discharges (*Medizinische Statistik der Krankenhäuser*).

Obesity prevalence among Swiss conscripts 2010-2012 (5.8 %) was slightly higher compared to the prevalence (3.6 %) detected among the small group of 165 men aged 15 to 29 years examined for the 2011 Swiss salt survey (however, 6.1 % of the same young men showed a substantially increased waist circumference (>102 cm) [34, 35]. Obesity prevalence (BMI ≥ 30 kg/m^2^) of 1,503 young men aged 15-24 participating in SHS in 2012 was 3.2 % and thus also lower compared with the conscripts, possibly due to the fact that SHS data were self-reported [25]. The levels of obesity (BMI ≥ 30 kg/m^2^) were slightly lower in Switzerland (5.8 %) compared to other European countries with mandatory conscription in 2010/2011 (Germany 8.5 %, Denmark 8.7 %, Austria 8.4 %) [52, 84]. In Austria, obesity prevalence among the conscripts varies between 6.9 % and 9.6 % between the three major regions of the country [52]. To our knowledge, this current study is the first in Switzerland, and generally the first based on conscription data, to analyse small-area variation in obesity prevalence on national level. For Switzerland, the highest resolution of regional analyses to date were cantons, major regions or districts [14, 55]. Lastly, the study confirms results of earlier studies on Switzerland and other countries showing higher BMI among individuals of lower socio-economic position [18–20].

Future studies could offer even higher spatial resolution of the analyses and use the main city of residence or even full residential addresses geocoded to point data instead of the data aggregated to postcodes. Furthermore, more conscription years could be included, which would allow use of spatio-temporal models [63, 85]. Future studies could explore non-linear methods of modelling continuous covariates and alternative, non-contiguity-based spatial weights such as road network connectivity as suggested by recent studies on mortality and life expectancy in Switzerland [61, 86]. They should also aim at capturing highest possible level of spatial reference of place of residence of the study participants in order to further investigate complex spatial patterns of OWOB. Lastly, and more generally - because of the on-going nature of conscription, the dataset together with spatial models could facilitate development of the monitoring system.

## Conclusion

The present study contributes to the understanding of spatial and socio-economic pattering of obesity in Switzerland. The results indicate large discrepancies in the risk of obesity among Swiss conscripts and could assist planning interventions on a small scale, irrespectively of administrative boundaries. Research on obesity and other branches of public health in Switzerland might benefit from leaving the usage of the common administrative boundaries behind.

## Additional file

Additional file 1: Figure S1. Distribution of postcode level estimated odds ratios (ORs) of obesity from unadjusted (top panel) and adjusted (bottom panel) models with various use of random effects. (PDF 18 kb)
