# Supplementary figures and images for: Finding big shots: small-area mapping and spatial modelling of obesity among Swiss male conscripts
Source: BMC Obes. 2016 Feb 18;3:10. doi: 10.1186/s40608-016-0092-6 (PMC4758017; doi:10.1186/s40608-016-0092-6)

Density of estimated odds ratios: Comparison of the models

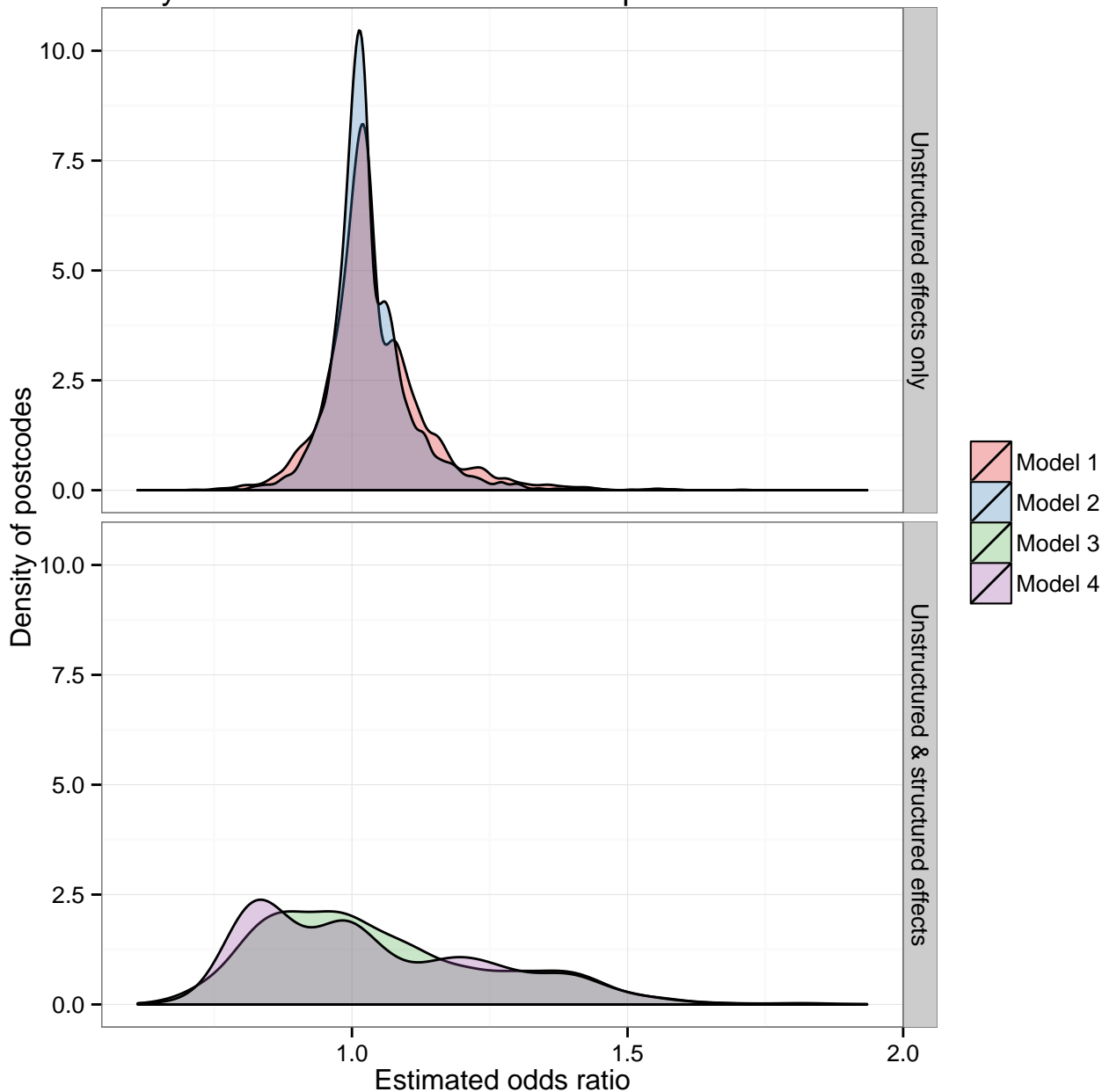

Supplement: Additional file 1: Figure S1. — Distribution of postcode level estimated odds ratios (ORs) of obesity from unadjusted (top panel) and adjusted (bottom panel) models with various use of random effects. (PDF 18 kb) [file 40608_2016_92_MOESM1_ESM.pdf]
